# Integrated transcriptomic and metabolomic analysis provides insights into cold tolerance in lettuce (*Lactuca sativa* L.)

**DOI:** 10.1186/s12870-024-05099-0

**Published:** 2024-05-23

**Authors:** Xiao Yang, Yingying Han, Guotao Huo, Guojun Ge, Shuping He, Xiao Yang, Li Zhang, Shiwei Wei, Lijun Luo

**Affiliations:** 1 Shanghai Agrobiological Gene Center, Shanghai, 201106 China; 2 Institute of Biothermal Science and Technology, School of Health Science and Engineering, University of Shanghai for Science and Technology, 516 Jungong Road, Shanghai, 200093 China; 3 Institute of Urban Agriculture, Chinese Academy of Agricultural Sciences, Chengdu National Agricultural Science and Technology Center, Chengdu, 610213 China; 4 Key Laboratory of Grain Crop Genetic Resources Evaluation and Utilization, Ministry of Agriculture and Rural Affairs, Shanghai, 201106 China

**Keywords:** Lettuce, Transcriptome, Metabolome, Flavonoids, Cold-stress

## Abstract

The popular leafy vegetable lettuce (*Lactuca sativa* L.) is susceptible to cold stress during the growing season, which slows growth rate, causes leaf yellowing and necrosis, and reduced yield and quality. In this study, transcriptomic and metabolomic analyses of two cold-resistant lettuce cultivars (GWAS-W42 and F11) and two cold-sensitive lettuce cultivars (S13K079 and S15K058) were performed to identify the mechanisms involved in the cold response of lettuce. Overall, transcriptome analysis identified 605 differentially expressed genes (DEGs), including significant enrichment of genes involved in the flavonoid and flavonol (*CHS*, *CHI*, *F3H*, *FLS*, *CYP75B1*, *HCT*, etc.) biosynthetic pathways related to oxidation–reduction and catalytic activity. Untargeted metabolomic analysis identified fifteen flavonoid metabolites and 28 other metabolites potentially involved in the response to cold stress; genistein, quercitrin, quercetin derivatives, kaempferol derivatives, luteolin derivatives, apigenin and their derivatives accumulate at higher levels in cold-resistant cultivars. Moreover, MYBs, bHLHs, WRKYs and Dofs also play positive role in the low temperature response, which affected the expression of structural genes contributing to the variation of metabolites between the resistant and sensitive. These results provide valuable evidence that the metabolites and genes involved in the flavonoid biosynthetic pathway play important roles in the response of lettuce to cold stress.

**Supplementary Information:**

The online version contains supplementary material available at 10.1186/s12870-024-05099-0.

## Introduction

Lettuce (*Lactuca sativa* L.) is a widely consumed salad vegetable with numerous health benefits [1]. Plants can grow in cool climates and have an ideal growing temperature of 15 to 20 ℃ [2]. Chilling injury occurs in lettuce at 0–8 ℃ and freezing injury occurs below 0 ℃ [3]. Freezing temperatures cause blistering and peeling of lettuce leaves, which leads to decay and rotting and also provides a pathway for pathogen entry [4]. Thus, cold has a significant impact on quality and yield in the lettuce industry [5]. The frequency and intensity of extreme weather events are increasing due to the severe effects of climate change in recent years. For example, prolonged freezing temperatures pose a significant challenge to the outdoor production of winter crops [6].

However, plants have developed a variety of adaptive strategies to cope with the constantly changing environment [7]. Some plants protect themselves from frost damage by constructing a complex barrier [8]. Additionally, a number of physiological and biochemical processes are induced in plants in response to cold stress, such as increased generation of reactive oxygen species (ROS) or antioxidants (such as polyphenols and flavonoids), or changes in the lipid content of membranes [9, 10].

Cold tolerance in plants is a complex trait involving a large number of genes and metabolites [11]. Analyses of the transcriptomes and metabolomes of *Poa crymophila* [12] and *Nicotiana tabacum* [13] have been conducted to determine the key metabolites, regulatory mechanisms, and candidate genes involved in the response of these plants to cold stress. Several studies have shown that multiple genes regulate the response of different organisms to low temperatures. The molecular mechanisms that regulate plant cold stress responses have been tentatively elucidated by analyzing differentially expressed genes (DEGs) (especially transcription factors) between cold-tolerant and cold-sensitive lines [14–16]. For example, transcription factors such as bHLH, NAC, C2H2, MYB, WRKY and AP2/ERF were identified in asparagus bean [17] and rice [18] exposed to low-temperature stress. In addition, two major families of transcription factors, AP2-EREBP and bHLH, show altered expression in *Magnolia wufengensis* during cold stress [19]. Moreover, metabolomic analysis showed that the levels of flavonols such as quercetin and dihydromyricetin were significantly increased in *Brassica napus* exposed to cold stress [20]. In addition, a variety of metabolites, especially flavonoids, were found to play important roles in the response of plants to cold stress [21].

The CBF/DREB1 gene family has been demonstrated to respond to a variety of environmental stresses in lettuce, including cold, heat, and salinity [4]. LsCBF5, LsCBF7, LsCBF11, LsCBF12 and LsCBF14 are up-regulated in response to cold and heat stress, suggesting that these genes regulate the response to abiotic stress and enable lettuce to adapt to a wider range of environmental conditions. Additionally, we have previously reported that several metabolites and transcription factors are involved in the response of lettuce to heat [22]. While these previous studies demonstrate that numerous metabolites play important roles in the cold stress response of lettuce, further research is needed to explore the precise functions of these metabolites, and there is a lack of research on the molecular mechanisms involved in the responses of lettuce to cold stress.

In this study, two cold-tolerant and two cold-sensitive lettuce cultivars were selected from 275 cultivars for further transcriptomic and metabolomic analysis. Bioinformatic analyses were performed to identify the major metabolites, regulatory pathways, and candidate genes involved in the response of the cold-tolerant and cold-sensitive cultivars to cold stress. We show that the flavonoid metabolic pathway is involved in the response of lettuce to cold stress. Overall, these mechanistic insights could be used to develop new biological regulators to protect lettuce plants from cold stress or to breed new lettuce varieties with improved cold tolerance.

## Results

### Evaluation of the cold tolerance of lettuce varieties

**Fig. 1:**
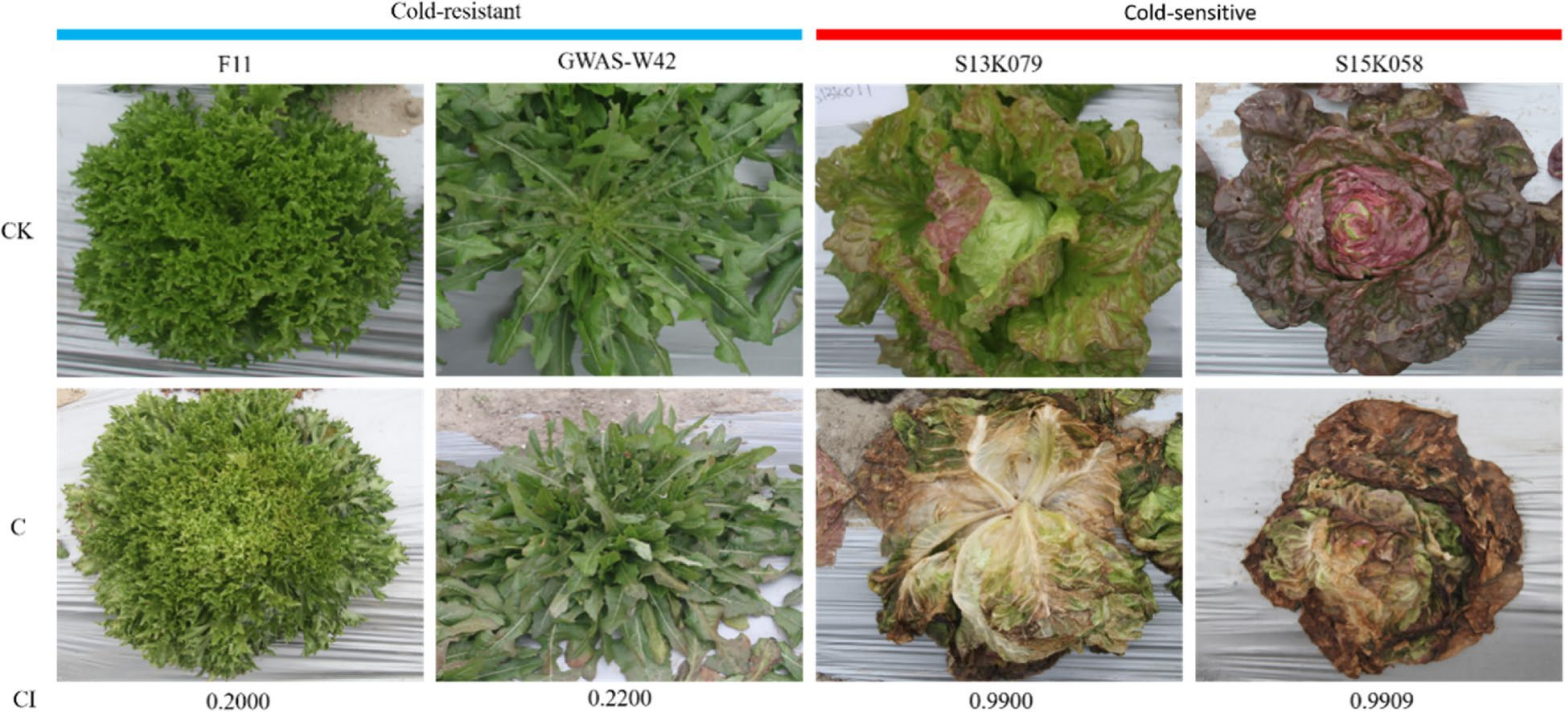
Field phenotypes of the two cold tolerant (F11 and GWAS-W42) and two cold sensitive (S13K079 and S15K058) lettuce varieties selected for this study. Note: ‘CK’ is the lettuce grown at normal temperatures, ‘C’ is the lettuce exposed to cold stress, and ‘CI’ is the chilling injury index

We evaluated the cold tolerance of 275 lettuce cultivars (unpublished data) and classified the cultivars into ten groups based on the mean ± standard deviation CI. This analysis identified eight cold tolerant varieties (level 2) and 12 cold sensitive varieties (level 10). Two extremely cold-resistant cultivars (F11, CI = 0.20 and GWAS-W42, CI = 0.22) and two extremely cold-sensitive cultivars (S13K079, CI = 0.99 and S15K058, CI = 0.99) were selected for further metabolomic and transcriptomic analysis. The CI values of the two cold-sensitive varieties were ~ 5 times higher than the CI values of the two cold-resistant varieties. The field phenotypes of the four varieties under cold stress are shown in Fig. 1. F11 and GWAS-W42 retained their original color and morphology after cold stress, while the leaves of the sensitive varieties turned brown and the plants were almost dead.

### Metabolomic analysis of metabolites involved in cold response

**Fig. 2:**
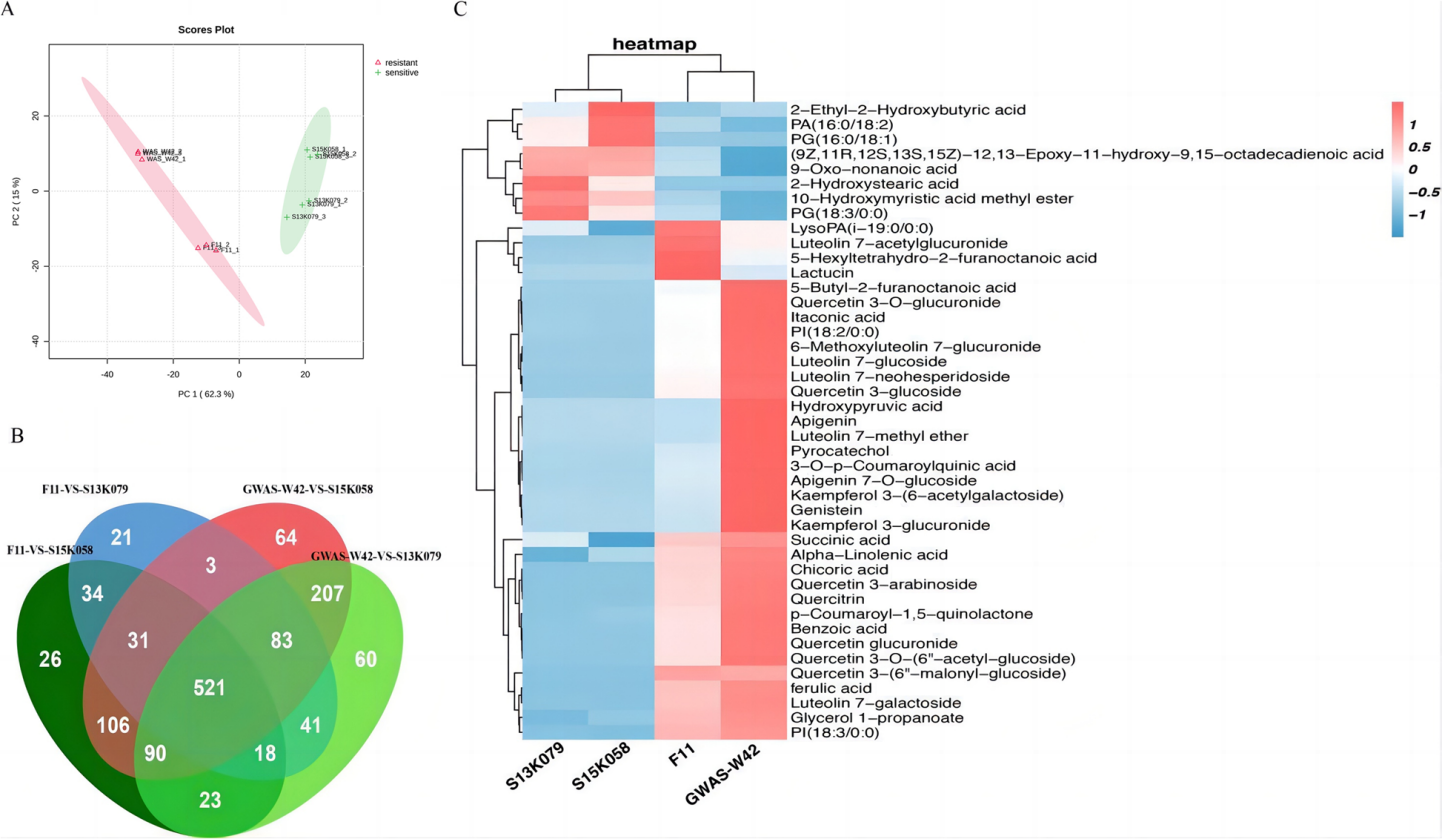
The analysis of the metabolites among the four lettuce varieties. **A** Principal component analysis (PCA) plots of differential metabolites in the two cold-tolerant and two cold-sensitive lettuce varieties (95% confidence interval) under cold stress. Green markers represent the cold-sensitive varieties; red markers represent the cold-tolerant varieties. **B** Venn diagram of the differential metabolite screen; **C** Heat map of the 43 significant differential metabolites between cold tolerant and cold sensitive lettuce varieties

We performed an untargeted metabolomic analysis of differential metabolites in the cold-tolerant and cold-sensitive lettuce varieties after exposure to cold stress. A total of 1433 metabolites were detected by High-Performance Liquid Chromatography Mass Spectrometer (HPLC–MS) in the four varieties. The abundances of these metabolites in each variety were analyzed by principal component analysis (PCA). As shown in Fig. 2A, the two cold-resistant cultivars (GWAS-W42 and F11) and two cold-sensitive cultivars (S13K079 and S15K058) clustered into two separate clusters. Two principal components were detected, suggesting that the differential metabolites play significant roles in the response of lettuce to cold stress. The first and second principal components explained 62.3% and 15% of the variance, respectively, and together explained 77.3% of the variance. These results indicate high similarity and repeatability within each group and that the metabolites are present at significantly different levels in the cold-resistant varieties and cold-sensitive varieties, confirming that the target materials are suitable for the next step of differential metabolite analysis.

We performed data filtering and standardized the metabolite abundances obtained by mass spectrometry. A total of 752 metabolites were differentially abundant between F11 and S13K079 (FDR < 0.05; Fig. S1A); 849 metabolites were differentially abundant between F11 and S15K058 (Fig. S1B); 1043 metabolites were differentially abundant between GWAS-W42 and S13K079 (Fig. S1C), and 1105 metabolites were differentially abundant between GWAS-W42 and S15K058 (Fig. S1D). Further screening of the four cultivars revealed that a total of 521 metabolites were significantly differentially abundant between the cold-tolerant and cold-sensitive cultivars (Fig. 2B).

However, since the chemical formulas derived from the first-order mass spectrometry information (mass-to-charge ratio, retention time) may correspond to a variety of substances, a secondary screening was performed to determine the second-order ion fragments of the mass spectra. Comparison of the theoretical ion fragments (using HMDB and lipid websites) and experimental ion fragment peaks of these 521 differentially abundant metabolites confirmed 43 differential metabolites. The abundances of these metabolites in the two cold-resistant and two cold-sensitive cultivars were filtered and standardized using the Metaboanalyst tool. Bidirectional clustering heat map analysis showed that the three duplicates of each cultivar clustered in one branch. Moreover, the two cold-resistant varieties and two cold-sensitive varieties also clustered in two separate branches, respectively (Fig. 2C). The most significant differential metabolites accumulated in the cold-resistant varieties. The corresponding metabolite information is shown in Table S1. These metabolites were classified into 13 categories; flavonoids represented the majority of the significant differential metabolites (*n* = 15), followed by fatty acyls (*n* = 9), and glycerophospholipids (*n* = 6; Fig. S2). The major flavonoids that accumulated significantly in the cold-resistant cultivars were apigenin, luteolin, quercetin, and derivatives of kaempferol; thus, the accumulation of these flavonoids may contribute to the ability of lettuce to withstand cold temperatures.

### Screening of DEGs involved in cold tolerance

**Fig. 3:**
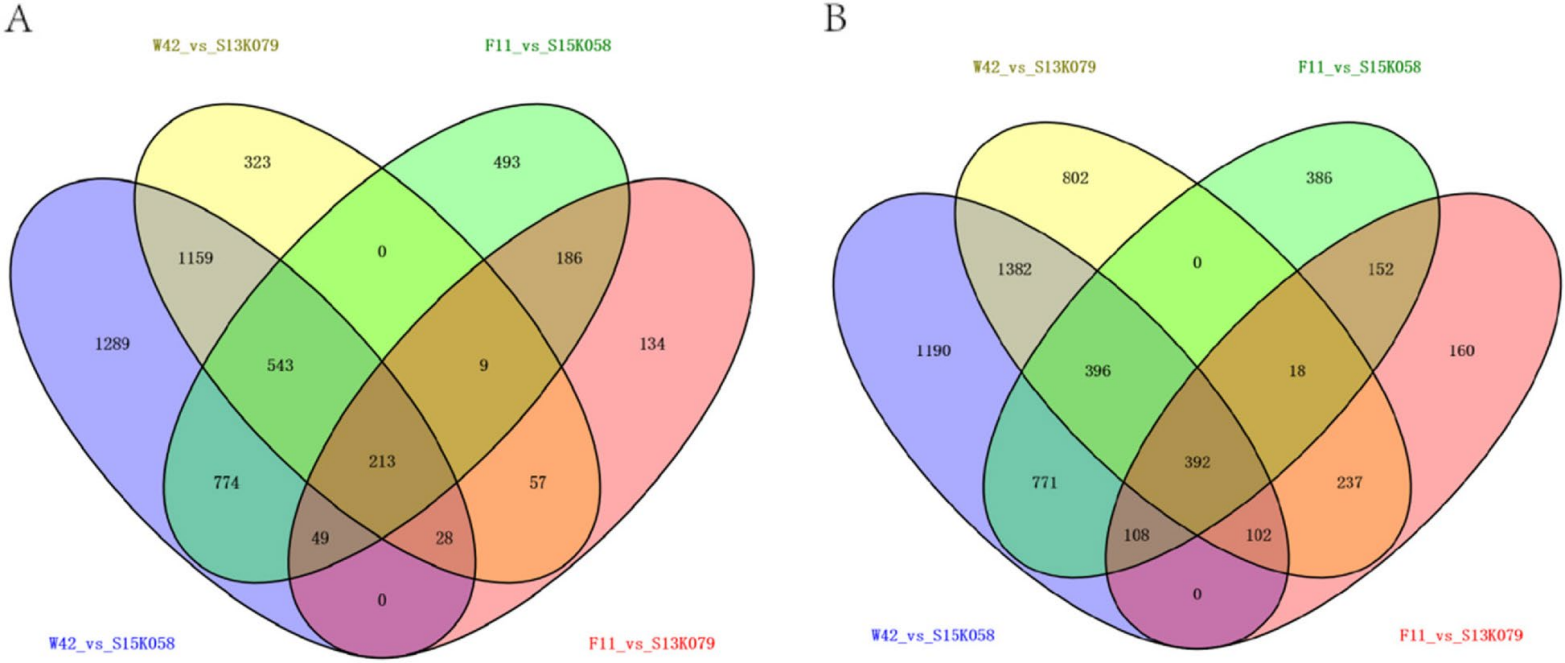
Venn diagrams of DEGs in lettuce exposed to cold stress. **A** Venn diagram of the 213 up-regulated DEGs in the two cold-resistant cultivars; **B** Venn diagram of the 392 down-regulated DEGs in the two cold-resistant cultivars; F11: F, S13K079: S13K, S15K058: S15K, GWAS-W42: W/W42

Transcriptome sequencing of these four varieties was also performed to reveal the potential genes and mechanisms. Twelve library reads were obtained; each library had approximately 40 million reads. After filtering, the number of reads varied from 36 to 43 million, representing an average of 91.6% of the total number of reads in all libraries (Table S2). The DEGs were selected based on using the criteria of a |log2FoldChange|> 1 and *P*-value < 0.05 as the threshold. Compared to F11 as the control, 1169 DEGs were upregulated and 676 DEGs were downregulated in S13K079 (Fig. S3A, Appendix 1) and 2223 DEGs were upregulated and 2267 DEGs were downregulated in S15K058 (Fig. S3B, Appendix 1). Using GWAS-W42 as the control, 3329 DEGs were upregulated and 2332 DEGs were downregulated in S13K079 (Fig. S3C, Appendix 1) and 4341 DEGs were upregulated and 4055 DEGs were downregulated in S15K058 (Fig. S3D, Appendix 1). Furthermore, we attempted to filter the DEGs between the cold-tolerant and cold-sensitive cultivars, which may help to reveal the molecular mechanisms of the response of lettuce to cold. The Venn diagram revealed a total of 605 DEGs between the cold-resistant and cold-tolerant cultivars: 213 DEGs were up-regulated and 392 DEGs were down-regulated in the cold-resistant cultivars (Fig. 3A and B).

From these 605 DEGs, several genes were randomly selected to verify their expression levels by qRT-PCR. Finally, genes with a significant difference in gene expression between cold tolerant varieties and cold sensitive varieties were identified (Fig. S4). The expression levels of these genes were consistent with the transcriptome results, indicating that high quality and accurate transcriptomic data were obtained in this study.

### Gene ontology(GO) and Kyoto Encyclopedia of Genes and Genomes(KEGG) enrichment analysis of terms involved in cold tolerance

To further investigate the biological pathways and functions of DEGs, we performed GO enrichment and KEGG analysis. The top 20 GO terms with the most significant enrichment were selected and are shown in Fig. S5. Most of the genes are involved in the biological process (BP) category; the oligosaccharide biosynthetic process was the most significantly enriched term in the BP category. The most notable term in the molecular function category is catalytic activity and the most notable term in the cell component (CC) category is integral component of membrane.

**Fig. 4:**
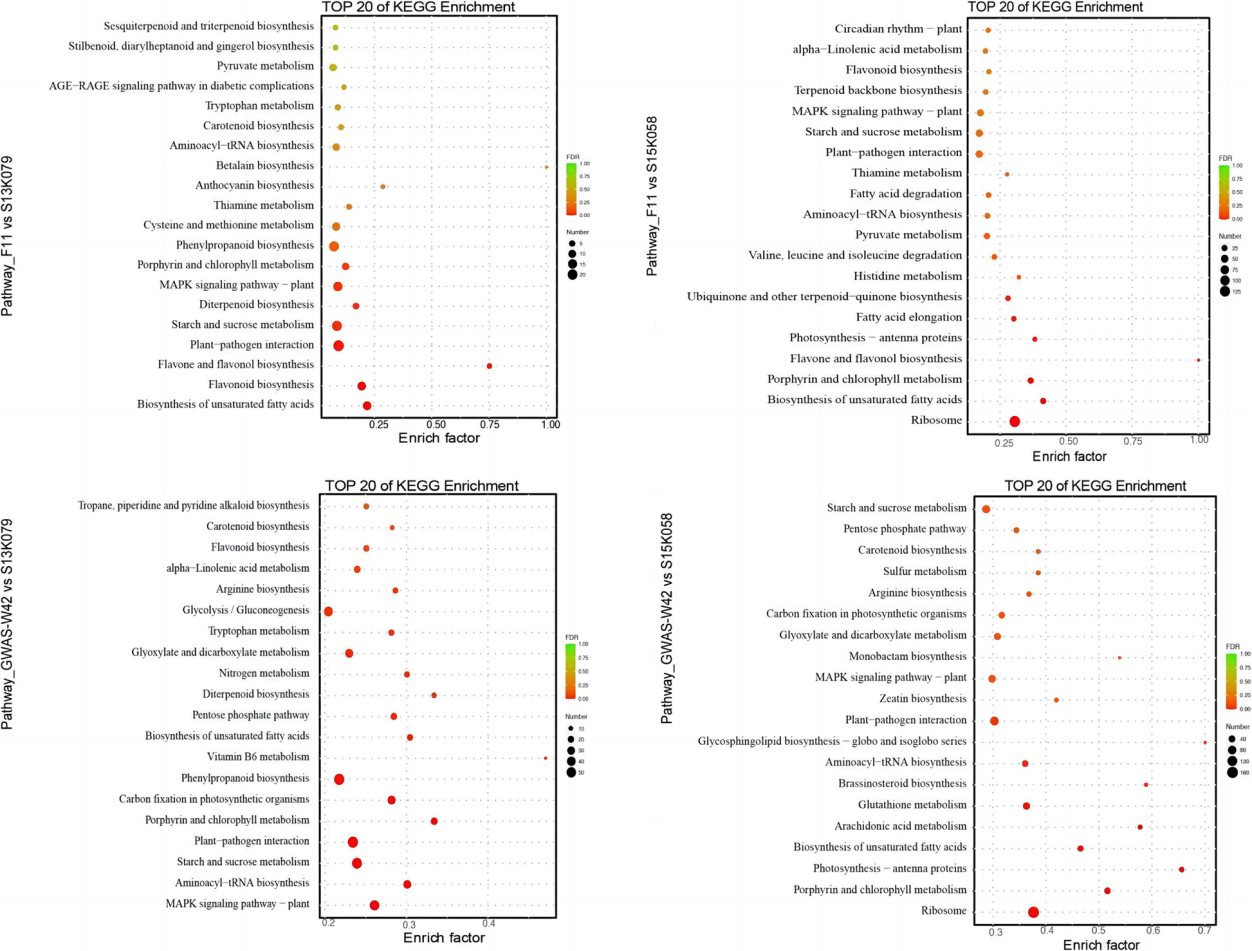
The pathway analysis in F11 vs. S13K079, F11 vs. S15K058, and GWAS-W42 vs. S13K079, GWAS-W42 vs. S15K058

We selected the top 20 pathways with the most significant enrichment in the KEGG enrichment analysis. As shown in Fig. 4, in F11 vs S13K079 group, the most enriched pathways are biosynthesis of unsaturated fatty acids, flavonoid biosynthesis, flavone and flavonol biosynthesis, plant-pathogen interaction, starch and sucrose metabolism. In the F11 vs S15K058 group, biosynthesis of unsaturated fatty acid biosynthesis, porphyrin and chlorophyll metabolism, flavone and flavonol biosynthesis, photosynthesis—antenna proteins, and ubiquinone and other terpenoid quinone biosynthesis were enriched. Additionally, in GWAS-W42 vs S13K079 group, MAPK signaling pathway – plant, aminoacyl-tRNA biosynthesis, starch and sucrose metabolism, porphyrin and chlorophyll metabolism, carbon fixation in photosynthetic organisms and phenylpropanoid biosynthesis were enriched. Moreover, porphyrin and chlorophyll metabolism, photosynthesis—antenna proteins, biosynthesis of unsaturated fatty acids, glutathione metabolism and brassinosteroid biosynthesis were enriched in GWAS-W42 vs S15K058 group.

### Analysis of weighted gene co-expression network (WGCNA) for genes associated with cold stress

**Fig. 5:**
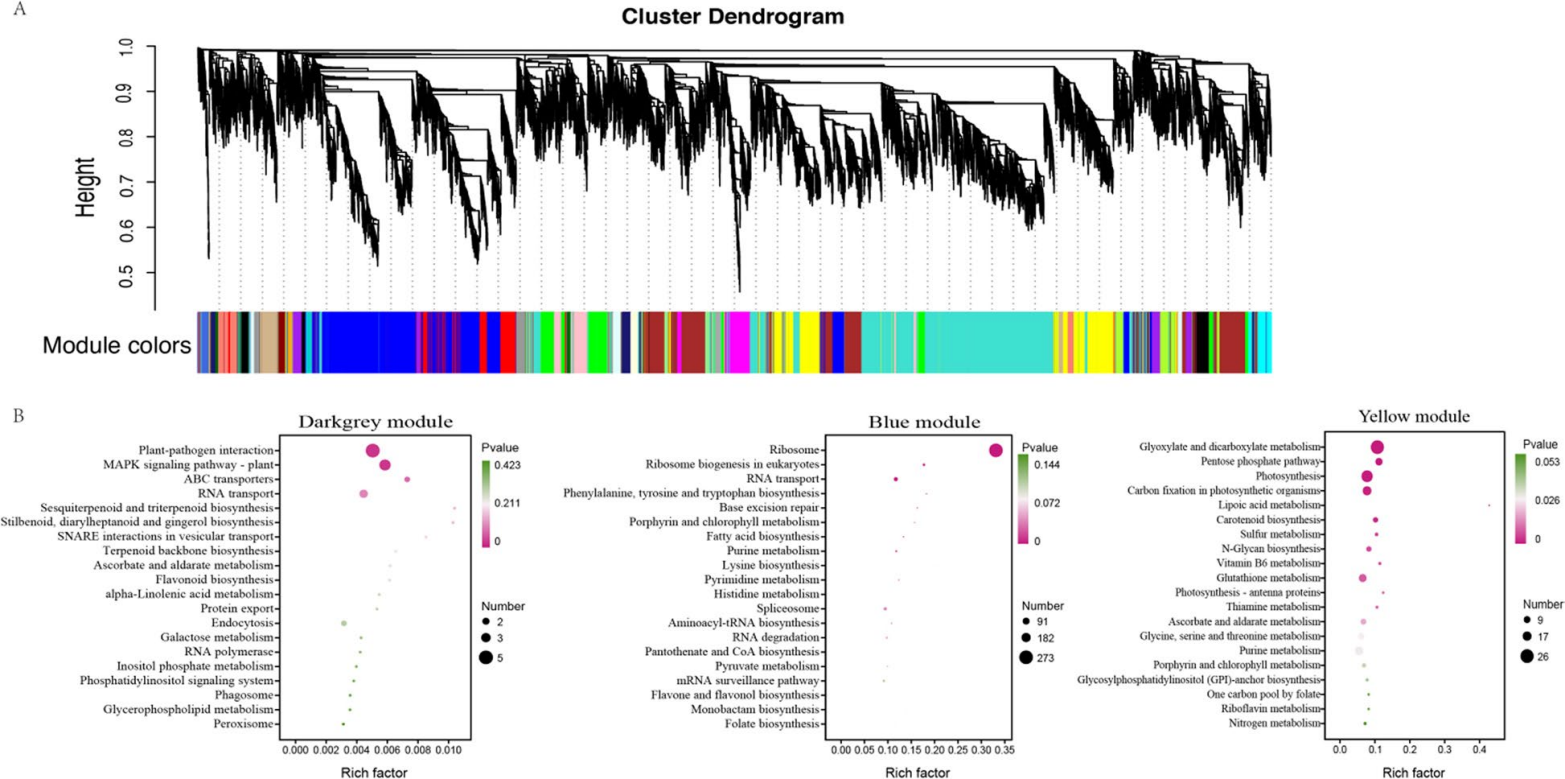
WGCNA analysis for genes related to cold stress response. **A** The cluster dendrogram of genes. Different colors were used to indicate the genes grouped into different modules in the dendrogram. The dendrogram was constructed using the optimal soft threshold. **B** The KEGG pathway enrichment of different modules with FDR < 0.1

WGCNA was performed to further investigate global transcriptomic organizations that may underlie genotype-specific responses to cold stress. The co-expression network was constructed using the optimal soft threshold, dividing the genes into different modules and performing the cluster dendrogram (Fig. 5A). From the results of WGCNA, 27 gene modules were identified, and marked with different colors. The modules with the highest potential associations with traits and phenotypes were identified by analyzing the correlation between module eigenvalues and specific trait and phenotype data. As a result, blue and yellow modules were positively correlated with flavonoid metabolites. Whereas, the darkgrey module was negatively correlated with flavonoid metabolites (Fig. S6). KEGG pathway enrichment analysis supported that blue module genes were mainly involved in ribosome and ribosome biogenesis in eukaryotes, while yellow module genes were related to glyoxylate and dicarboxylate metabolism, pentose phosphate pathway and photosynthesis pathway. The darkgrey module genes were enriched in plant-pathogen interaction and mitogen-activated protein kinase (MAPK) signaling pathway – plant pathways (Fig. 5B). Additionally, we also identified flavonoid biosynthesis, flavone and flavonol biosynthesis as enriched in darkgrey and bule modules, respectively.

### Metabolic pathway mapping and transcript factor identification

**Fig. 6:**
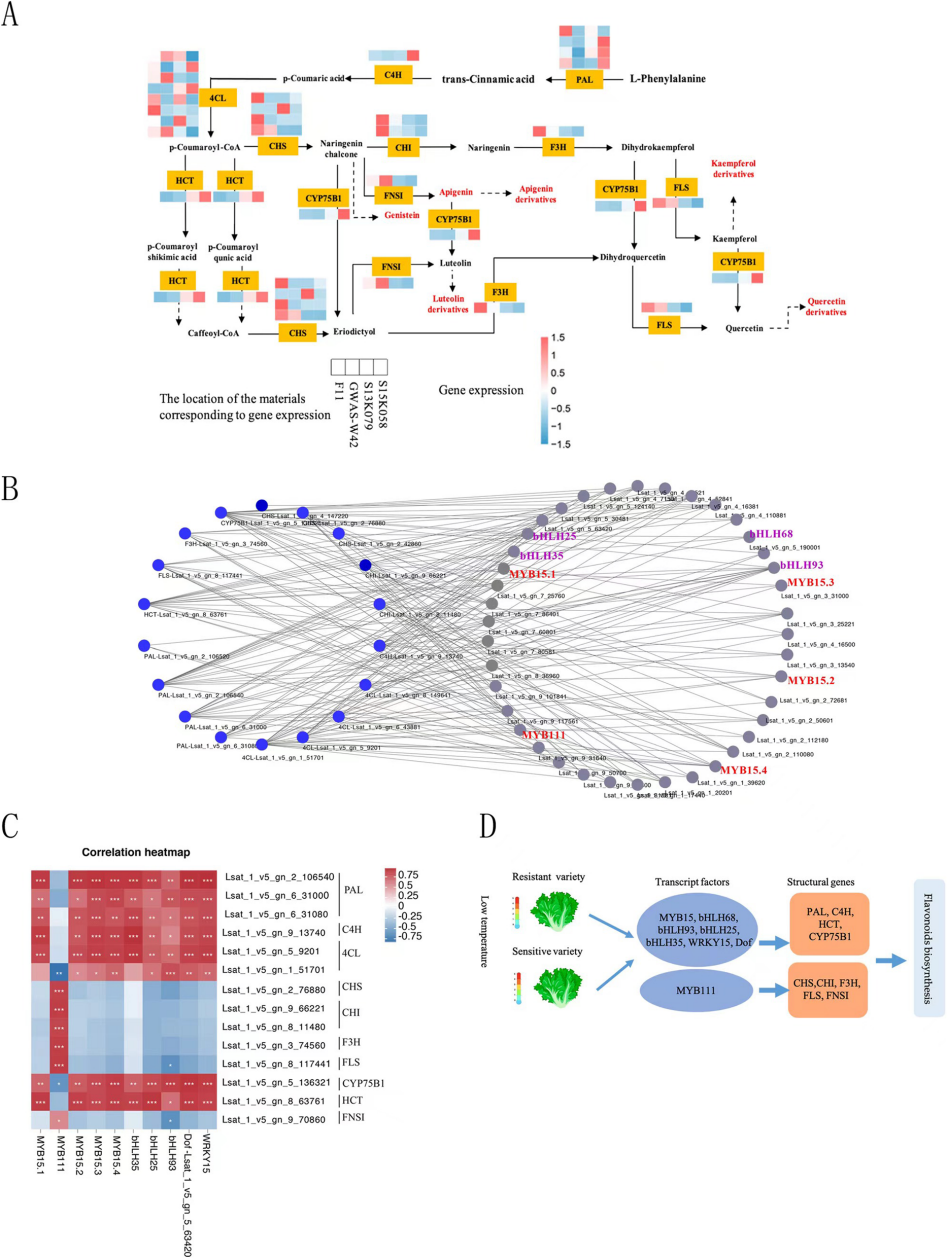
Transcript factors associated with flavonoid biosynthesis. **A** Map of pathways associated with the DEGs in lettuce under cold stress. Yellow boxes represent DEGs or enzymes of interest. Gene expression data were normalized based on the mean expression value of each gene in all samples analyzed. Red and blue boxes indicate high and low expression levels, respectively, for each gene. **B** Network of transcript factors (blue nodes) and structural genes of flavonoid biosynthesis (grey nodes). **C** Pearson’s correlation coefficients between the expression levels of flavonoid biosynthesis genes and the expression levels of transcript factors. Blue cells indicate low Pearson’s correlation value. Red cells indicate high Pearson’s correlation value. Asterisks indicate significance at *p* < 0.05. **D** Working model of how lettuce responds to low temperature. Low temperature triggers the expression of *MYB15*, *bHLHs*, *WRKYs*, *Dofs* and induces the structural genes involved in flavonoid biosynthesis, including *PAL*, *C4H*, *CYP75B1,* and *HCT*. And *MYB111* induced the expression of *CHS, CHI, F3H, FLS, FNSI*

Metabolite analysis indicated that flavonoids represented the majority of the differential metabolites involved in cold tolerance in lettuce. In parallel, KEGG enrichment also showed that flavonoid pathway genes were among the significantly enriched differential genes. Thus, the expression patterns of 18 genes encoding 9 biosynthetic enzymes in this pathway were extracted from our transcriptome (Fig. 6A). Phenylalanine ammonia-lyase (PAL), cinnamate 4-hydroxylase (C4H) and 4- coumarate: coenzyme A ligase (4CL), which are responsible for the biosynthesis of flavonoid precursor, show no significant expression change in expression in these four cultivars. Three (Lsat_1_v5_gn_4_147220, Lsat_1_v5_gn_2_42860 and Lsat_1_v5_gn_2_76880) of the four CHS members were highly expressed in F11 and GWAS-W42 compared with the sensitive cultivars. Additionally, two CHI (Lsat_1_v5_gn_9_66221 and Lsat_1_v5_gn_8_11480) genes were also highly expressed in the resistant varieties. F3H (Lsat_1_v5_gn_3_74560) and FNSI (Lsat_1_v5_gn_9_70860) showed the similar expression pattern. These genes might contribute to the significant accumulation of the flavonoids genistein, quercitrin, quercetin, kaempferol, apigenin and its derivatives, and luteolin derivatives in the cold-resistant varieties (GWAS-W42, F11; Fig. 6A). The genes listed above were up-regulated in the cold-resistant varieties (GWAS-W42, F11) and are related to the functions oxidation–reduction and catalytic activity functions, with the exception of HCT and CYP75B1.

Therefore, we speculate that the high expression of the *CHS*, *CHI*, *F3H*, *FLS* and *FNSI* genes leads to the accumulation of kaempferol and quercetin in the cold-resistant varieties. Kaempferol and quercetin can be converted into various derivatives, such as kaempferol 3-glucuronide, kaempferol 3-(6-acetylgalactoside), quercetin 3-O-glucuronide, quercetin 3-glucoside, quercetin 3-O-(6''-acetyl-glucoside), quercetin glucuronide, and quercetin 3-arabinoside. CHS metabolizes other related metabolites to naringenin chalcone, which is then converted by FNSI to apigenin and various derivatives. In addition, high expression of *FLS* and *FNSI* may result in significant accumulation of luteolin derivatives (such as luteolin 7-acetylglucuronide, luteolin 7-galactoside, luteolin 7-glucoside, luteolin 7-methyl ether, and luteolin 7-neohesperidoside) in the cold-resistant varieties.

Next, we investigated the general regulatory pathways of flavonoid biosynthesis and how flavonoid-related metabolism differed in these four cultivars. Based on gene expression, an interactive network of those transcript factors and structural genes involved in flavonoid biosynthesis was established (Fig. 6B and C). The results showed that *MYBs*, *bHLHs*, *WRKYs*, and *Dofs* were tightly correlated with the genes in the flavonoid biosynthesis pathway. In detail, 10 MYBs regulate 14 genes encoding 9 enzymes in the flavonoid biosynthesis pathway. *MYB111* (MYB111-like) were positively correlated with structural genes including *CHS,CHI, F3H, FLS, FNSI*, negatively correlated with *CYP75B1* and *4CL* (Lsat_1_v5_gn_1_51701). While, the other transcript factors including *MYB15* (MYB15-like), *WRKY15* and Dof (Lsat_1_v5_gn_5_63420) were positively correlated with *PAL, C4H, 4CL, CYP75B1 and HCT*. 8 bHLH transcript factors were closely associated with 13 genes encoding 7 enzymes in the flavonoid biosynthetic pathway. Also, 5 WRKYs and 9 Dofs were positively correlated with structural genes in flavonoid biosynthesis pathway. A possible mechanism of low temperature response between the resistant and sensitive cultivars was shown in Fig. 6D. In this model, MYB15, MYB111, and other MYB-related genes were more highly expressed in resistant varieties. In addition, the bHLHs, WRKYs and Dofs were also plays positive role in the low temperature. And low temperature triggers the expression of *MYB15*, *bHLHs*, *WRKYs*, *Dofs* and induced the structural genes involved in flavonoid biosynthesis including *PAL, C4H, CYP75B1* and *HCT*. And *MYB111* induced the expression of *CHS,CHI, F3H, FLS, FNSI*. Collectively, these results provide valuable information on the molecular mechanisms that contribute to the response of lettuce to cold stress.

## Methods

### Investigation of chilling injury

Seeds of 275 lettuce cultivars were obtained from the lettuce germplasm bank of the Shanghai Agricultural Gene Center, sown in plastic pots, and the seedlings grown in a greenhouse as described in our previous research [23]. All plants were cultivated under normal conditions until almost harvest, and then subjected to low-temperature stress for 2 weeks; in addition, the temperature remained below 0 ℃ for 5 days (from December 29, 2020 to January 2, 2021) in Zhuanghang Town, Fengxian District before the investigation (Fig. S7). The atmospheric temperature data were downloaded from China Weather Network (http://www.weather.com.cn/). First, the chilling injury (CI) index, which reflects the area of damaged leaves, was determined on January 4, 2021, as described by Liu and Li [24, 25]; the collection criteria are listed in Supplementary Table S3. Plants were sampled the next day for the metabolic analyses; then, the CI values were used to grade the most extreme lettuce material, and the criteria were according to the mean value (X) and standard deviation (σ), with grade 1 < X-2σ and grade 10 ≥ X + 2σ, with a difference of 0.5σ between each grade [26].

$$\mathrm{Chilling\ injury\ index }\left({\text{CI}}\right)=\left(\sum \left(\mathrm{Number\ of\ per\ level}\times {\text{level}}\right)\right)/\left(\mathrm{The\ highest\ level}\times \mathrm{Total\ number\ of\ plants}\right)$$

Samples for transcriptome and metabolome analysis were collected the day after chilling; approximately 16 plants were collected for each cultivar. All transcriptome and metabolome samples in this study were collected after cold injury to compare the cold-resistant and cold-sensitive varieties. The cold-resistant materials were sampled at the position where the tip of the leaf avoided the vein. The leaves of most of the cold-sensitive materials were severely damaged, so the leaves in the best condition were sampled.

### Metabolite profiling

#### HPLC–MS analysis

Lettuce samples (200 mg) of the cold-resistant and two cold-sensitive materials (three biological replicates per material) were ground to powder using liquid nitrogen, extracted with 1 mL methanol/water (4:1 v/v) [23], and untargeted metabolite analysis was performed by UPLC-IMS-QTOF-MS.

The LC conditions [27] were as follows: column (BEH C18 1.7 µm filler), 2.1*100 mm (precolumn); column temperature: 45 °C; flow rate: 0.4 mL/min; mobile phase A: 0.1% formic acid water, mobile phase B: 0.1% formic acid in acetonitrile; gradient elution conditions: 0‒3 min, 95% A and 5% B; 3–10 min, 80% A and 20% B; 10–12 min, 0% A and 100% B; 12–15 min, 0% A and 100% B; 15–19 min, 5% A and 95% B; injection volume: 1 μL. MS conditions: acquisition mode: MSE; ionization mode: ESI negative; capillary voltage: 2 kV (negative), cone voltage: 40 V; desolvation temp: 450 ℃; desolvation gas: 900 L/h; cone gas: 50 L/h; source temp: 115 ℃; acquisition range: 50 to 1000 m/z; scan rate: 0.2 s; collision energy: 6 eV/20 ~ 45 eV.

#### Metabolite identification

Putative metabolites were detected on the basis of a mass error < 5 ppm. Metabolites from LC/MS were putatively detected by comparing accurate masses and MS2 fragments with online reference databases (HMDB and Lipid) [28, 29] and bibliographies. At least two major fragments had to be detected.

#### Data processing

A data matrix (Appendix 2), containing the names of the compounds and their raw abundances in each of the three biological replicates for each sample, was generated and output from the embedded software of the UPLC-IMS-QTOF-MS (Progenesis QI). Differential metabolite screening was then performed using Metaboanalyst based on a false discovery rate (FDR) < 0.05.

### RNA-Sequencing (RNA-seq)

#### RNA extraction and sequencing

RNA-seq was performed by Personal Biotechnology Co., Ltd. Shanghai, China. Total RNA was extracted from triplicate samples of the freshly collected lettuce leaves using TRIzol reagent (Invitrogen, Carlsbad, CA, USA) according to the manufacturer’s protocol. The quality of the total RNA samples was evaluated using an Agilent 2100 Bioanalyzer. After RNA extraction, purification, and library construction, the libraries were sequenced by next-generation sequencing (NGS) on an Illumina sequencing platform [30]. The sequencing data volume was 6 G (number of bases), and the reference genome was downloaded from https://lgr.genomecenter.ucdavis.edu/. The generated library sequencing data have been deposited at the NCBI (https://www.ncbi.nlm.nih.gov/) with the accession number PRJNA759325.

#### Analysis of DEGs

DESeq was used for differential gene expression analysis [31]. The screening conditions for DEGs were a |log2FoldChange|> 1 and a *P*-value < 0.05 [32]. Bidirectional clustering was used for cluster analysis. Distances were calculated using the Euclidean method, and clustering was carried out using Complete Linkage (Shanghai Personalbio). The criterion for significant enrichment of differential genes was a *P* value < 0.05. The databases PlantTFDB (Plant Transcription Factor Database) [33] and AnimalTFDB (Animal Transcription Factor Database) [34] were used for transcription factor prediction.

#### Fluorescent quantitative PCR (qRT-PCR) analysis

Quantitative real-time PCR (qPCR) analysis was performed to further validate the RNA-Seq data using primer sets specific for the target genes (Table S4). The RNA samples for qRT-PCR were the same as for transcriptomic analysis. The cDNA synthesis was conducted using the cDNA Synthesis SuperMix kit (TransGen Biotech, Beijing, China) according to the manufacturer’s instructions. qRT-PCR experiment was performed using the TransGen’s PerfectStart Green qPCR SuperMix kit. Perform qRT-PCR using the PerfectStart Green qPCR SuperMix (TransGen Biotech) on the CFX96TM Optics Module (Bio-Rad Laboratories). Lettuce Actin-7 (LOC111882438) gene was selected as the reference gene for normalization. The reaction mixture consisted of 10 µL of 2 × mix, 1 µL each of forward and reverse primers, 1 µL of cDNA template, and 7 µL of ddH_2_O. Three technical replicates were performed for each biological replicate during qPCR analysis. Primers for qPCR were designed using Sangon Biotech website (Shanghai, China) (https://www.sangon.com/) and synthesized by BioSune Company (Shanghai, China).

The qPCR thermal cycling conditions included initial denaturation at 94 ℃ for 30 s, followed by 40 cycles of amplification at 94 ℃ for 5 s, 55 ℃ for 15 s, and 72 ℃ for 10 s. The melting curve analysis was performed with default settings: 65 ℃ for 5 s, 65 ℃ to 95 ℃ (0.5 ℃/s). Fluorescence was measured at the end of each cycle for quantification (Cq), and three technical replicates (*N* = 3) were pooled. Amplification, detection, and data analysis were conducted using the CFX96™ Optics Module (Bio-Rad, America). The 2^−ΔΔc^t method [35] was used to calculate the relative gene expression level.

### Analysis tools

The transcriptomic and metabolic data were analyzed following our previously described method [36]. Metaboanalyst was used for PCA analysis, metabolite heat map analysis, and analysis of differential metabolites between cultivars. Venn diagrams, gene heat maps, differential gene GO enrichment maps and KEGG enrichment maps were generated using genescloud tools (Personalbio). All other line charts and bar graphs were generated using GraphPad (version, 8.4.2). Significance tests were performed using one-way ANOVA.

### Weighted gene co-expression network analysis

The R package (version 3.6) WGCNA (Weighted gene co-expression network analysis) was used to discover the co-expression network and module [37]. In order to find correlations between the total gene expression levels of biological replicates in the RNA-seq experiment (R2 > 0.8), Pearson’s correlations were performed. Genes encoding transcription factors and flavonoid-related enzymes that were positively correlated with flavonoid content with a Pearson correlation coefficient greater than 0.8 were used to construct the correlation network using the same method with the COV cutoff of 0.1. The networks were visualized using Cytoscape v.3.8.0 [38].

### GO and KEGG enrichment analysis

To gain insight into the functional and pathway enrichment of DEGs between cold-tolerant and sensitive cultivars, we performed GO and KEGG enrichment analyses. Enrichment results were presented for the top 20 GO terms and KEGG pathways. The significance of enrichment was defined as a *P*-value < 0.05 and FDR < 1. In the subsequent KEGG enrichment analysis of the WGCNA module, the significance criterion for enrichment was set as FDR < 0.1.

## Discussion

### Accumulation of flavonoids and fatty compounds is associated with cold resistance in lettuce

In this study, metabolomic analysis detected 43 differential metabolites between two cold-tolerant and two cold-sensitive lettuce varieties, of which 36 metabolites were significantly enriched in the cold-resistant varieties. Interestingly, 15 flavonoids were significantly enriched in the cold-tolerant varieties compared to the cold-sensitive varieties, including genistein, quercitrin, quercetin derivatives, kaempferol derivatives, luteolin derivatives, and apigenin and its derivatives; these findings are consistent with the results of previous studies [39]. Flavonoids have previously been implicated in the response of plants to cold stress and heat stress [36, 40]. For example, HPLC–MS analysis showed that the luteolin content of pepper increased after low temperature treatment [41]. Flavonol metabolites (catechin, epicatechin, rutin, and quercetin) also accumulated significantly after cold stress in *T. hemsleyanum*, and related genes were also up-regulated [42]. In *Arabidopsis thaliana*, exposure to low temperature significantly increased the accumulation of quercetin derivatives, kaempferol and other polyphenols. Knockout of the flavonol biosynthesis-associated transcription factors MYB11, MYB12 and MYB111 in *Arabidopsis* increased leaf sensitivity to low temperature [43]. In parallel, our transcriptomic analysis detected several DEGs involved in the flavonoid biosynthesis pathway. For example, CHS, CHI, F3H, FLS and FNSI were upregulated in the cold-resistant cultivars, and are likely to lead to the accumulation of quercetin derivatives and kaempferol derivatives. However, HCT was down-regulated in the cold tolerant varieties. CtHCT3 expression was found to be significantly and negatively correlated with the kaempferol content in safflower grown under different light intensities [44]. In our previous results, we also observed the accumulation of flavonoid compounds after exposure to heat stress. Luteolin-7 glucoside were found to accumulate significantly in both heat and cold stress response in lettuce. Therefore, our work suggests that the accumulation of flavonoid compounds, especially quercetin derivatives and luteolin derivatives, may play an important role in the response of lettuce to both cold stress and heat stress.

Fatty compounds (fatty acyls) were the second most abundant category of metabolites in the metabolomic analysis after cold stress, which is different from heat stress response [36]. Acyl carrier protein (ACP) is an important component in FA biosynthesis, three ACPs (Lsat_1_v5_gn_4_38561, Lsat_1_v5_gn_4_84020, Lsat_1_v5_gn_4_84821) were up-regulated in cold-tolerant varieties. GO enrichment revealed that the terms integral component of membrane and intrinsic component of membrane were significantly enriched in the cold-tolerant lettuce varieties. Lsat_1_v5_gn_2_8861 (GDSL esterase/lipase, GDSL), a DEG that was verified to be up-regulated in the cold-tolerant varieties, was previously associated with cold tolerance in apricot trees [45]. This is consistent with reports that low temperature can induce stomatal closure, changes in membrane lipid composition, and accumulation of abscisic acid (ABA) and proline in plants [46]. The leaves of the cold-sensitive lettuce were extensively damaged by cold stress, which would lead to membrane changes. In addition, ABA has been reported to enhance cold tolerance by increasing the activity of antioxidant enzymes [47]. Lsat_1_v5_gn_8_14780 (Delta-1-pyrroline-5-carboxylate synthase, P5CS1) were up-regulated in cold tolerance cultivars in association with proline accumulation [48]. Additionally, ABA biosynthesis-related genes were also up-regulated in cold-resistance varieties compared with sensitive varieties, including Lsat_1_v5_gn_5_120740 (putative 9-cis-epoxycarotenoid dioxygenase 3, NCED), Lsat_1_v5_gn_9_111181 (Zeaxanthin epoxidase, ZEP) [49]. In conclusion, the DEGs and differential metabolites detected in this study provide a knowledge base for the breeding of cold-resistant lettuce.

### DEGs between cold-tolerant and cold-sensitive lettuce varieties

Among the approximately 42 million reads, a total of 605 significant DEGs (213 up-regulated and 392 down-regulated) were detected in the cold-resistant varieties (F11 and GWAS-W42) compared with the cold-sensitive varieties (S13K079 and S15K058). This result is similar to that of Wei et al. [50]. In lettuce, suggesting that different cultivars have similar responses to abiotic stress treatment. Additionally, this finding is consistent with the cold stress responses of other species, including *potato* [51] and *Prunus persica* [52]. GO classifications of the DEGs in this study revealed enrichment of several functional categories, mainly ‘oligosaccharide biosynthetic process’, ‘plastid transcription’, ‘oxidation–reduction process’, ‘catalytic activity’ and ‘protein serine/threonine kinase activity’. KEGG pathway analysis revealed that the principal enriched pathways included flavonoid biosynthesis, flavone and flavonol biosynthesis, starch and sucrose metabolism, phenylpropanoid biosynthesis, and the MAPK signaling pathway. Overall, this comprehensive transcriptomic analysis of cold-tolerant and cold-sensitive cultivars revealed a number of DEGs that play potentially important roles in the cold stress response of lettuce.

### Transcription factors contribute to cold resistance in lettuce

Transcription factors play an important role in abiotic response processes. In this study, the MYBs, bHLHs, WRKYs, and Dofs have been identified that were associated with low temperature response. For example, WRKYs have been reported to play a role in abiotic stress in various plants. In *Arabidopsis*, overexpression of AtWRKY25, AtWRKY26, and AtWRKY30 increased resistance to heat and salt [53, 54], and overexpression of AtWRKY46, AtWRKY54, and AtWRKY70 increased resistance to drought [55]. Additionally, overexpression of GmWRKY21 in *soybean* increased cold stress tolerance in transgenic *Arabidopsis* plants. GmWRKY54 confers salt and drought tolerance, while overexpression of GmWRKY13 increased sensitivity to salt and mannitol stress in transgenic *Arabidopsis* plants [56]. In parallel, six WRKY transcript factors were identified that were differentially expressed during heat stress in lettuce, suggesting that WRKYs play a role in heat stress response. In this study, our results also showed that five WRKY genes (Lsat_1_v5_gn_7_25760, Lsat_1_v5_gn_7_60801, Lsat_1_v5_gn_2_110080 Lsat_1_v5_gn_3_25221, and Lsat_1_v5_gn_5_124140) are involved in the cold stress response in lettuce. Additionally, bHLH and MYB/MYB-related transcription factors have also been reported to play important roles in abiotic stress tolerance. For example, OsMYB3R-2 was shown to mediate cold stress-related signal transduction and regulate stress response genes involved in the cell cycle or the derived DREB/CBF pathway, thereby enhancing cold tolerance in rice [57]. It has also been reported that MYB15 positively regulates cold tolerance in *tomato* through the CBF pathway [58]. In this study, we identified 4 MYB15 (Lsat_1_v5_gn_6_82640, Lsat_1_v5_gn_3_120520 Lsat_1_v5_gn_3_39080 and Lsat_1_v5_gn_3_39020) genes that showed higher expression levels in resistance, which were also closely related to structural genes in flavonoid biosynthesis pathway. The result is consistent with previous reports showing the positive role of MYB15 in cold response. Additionally, BcMYB111 was found to regulate flavonol synthesis by binding directly to the promoters of BcF3H and BcFLS1, resulting in flavonol accumulation [59]. In lettuce, the LsMYB111 (Lsat_1_v5_gn_9_181) was also been identified showing differential expression levels under low temperature between resistance and sensitive varieties, similar to structural genes including 4CL (Lsat_1_v5_gn_1_51701), CHS-Lsat_1_v5_gn_2_76880, CHI (Lsat_1_v5_gn_9_66221), CHI (Lsat_1_v5_gn_8_11480), F3H (Lsat_1_v5_gn_3_74560), and FLS (Lsat_1_v5_gn_8_117441), indicating their important role in cold response in lettuce. Moreover, we also demonstrated that bHLH transcript factors might function in cold stress response. OrbHLH001 was been reported to increase the tolerance of transgenic *Arabidopsis thaliana* to freezing stress [60]. AtbHLH68 [61] transcription factor was also shown to contribute to drought stress tolerance in *Arabidopsis thaliana*. In this study, bHLHs, including bHLH68, bHLH93, bHLH25, bHLH35, etc., were enriched in the cold-resistant varieties, indicating that these genes may also contribute to cold tolerance in lettuce. Thus, these transcript factors function by binding to structural genes to induce the flavonoid biosynthesis pathway to be activated, resulting in the accumulation of metabolites.

## Conclusion

The objective of our research was to reveal the important metabolites that play significant roles in cold tolerance in lettuce. We also used transcriptomics to further explain the accumulation of metabolites. Two cold-tolerant (CI: 0.20 and 0.22) and two cold-sensitive (CI: 0.99) lettuce cultivars were selected from 275 cultivars exposed to natural cold stress for transcriptomic and metabolomic analyses. Enrichment analysis and pathway mapping of the 43 differential metabolites revealed distinct differences in the starch and sucrose, fatty acid, and flavonoid biosynthetic pathways between the cold-tolerant and cold-sensitive cultivars. Importantly, several of the significantly enriched pathways are associated with flavonoids, such as flavone and flavonol biosynthesis; the largest number of differential metabolites were flavonoids (*n* = 15), followed by fatty acyls (*n* = 9), and glycerophospholipids (*n* = 6). Apigenin, luteolin, quercetin and kaempferol derivatives were the major flavonoids that accumulated significantly in the cold-resistant cultivars. Additionally, a total of 605 significant DEGs were identified in the cold-resistant cultivars (F11 and GWAS-W42) when compared to the cold-sensitive cultivars (S13K079 and S15K058). Interestingly, 9 of the DEGs (CHS, CHI, F3H, FLS, CYP75B1 and FNSI) may contribute to the significant accumulation of flavonoids observed in the cold-resistant varieties.

### Supplementary Information

Supplementary Material 1: Fig. S1. Screening of metabolites of different cold tolerant lettuce material response to cold stress. Fig. S2. Classification of 43 metabolites from different cold tolerant lettuce material response to cold stress. Fig. S3. Screening of DEGs in four lettuce varieties. Fig. S4. qRT-PCR validation of partial differential lettuce gene. Fig. S5. GO enrichment analysis of different expression genes from different cold tolerant lettuce material response to cold stress. Fig. S6. Partitioning Differential Genes into 27 Gene Modules Using WGCNA. Fig. S7. The temperature change during lettuce growth

Supplementary Material 2: Table S1. Specific information of 43 differential metabolites under cold stress in lettuce. Table S2. Summary of the RNA-seq data collected from lettuce. Table S3. Classification standard 1 for cold injury investigation of lettuce. Table S4. List of primers used in this study.

Supplementary Material 3: Appendix 1. Differentially expressed genes between cold-tolerant and sensitive materials.

Supplementary Material 4: Appendix 2. Raw abundance data of metabolites.

## Data Availability

The datasets generated and/or analysed during the current study are available in the National Center for Biotechnology Information (NCBI) repository, https://www.ncbi.nlm.nih.gov/ OR PRJNA759325.
