# Supplementary material for: Integrated transcriptomic and metabolomic analysis provides insights into cold tolerance in lettuce (Lactuca sativa L.)
Source: BMC Plant Biol. 2024 May 23;24:442. doi: 10.1186/s12870-024-05099-0 (PMC11112944; doi:10.1186/s12870-024-05099-0)
Supplement: Supplementary file 1 — Supplementary Material 1: Fig. S1. Screening of metabolites of different cold tolerant lettuce material response to cold stress. Fig. S2. Classification of 43 metabolites from different cold tolerant lettuce material response to cold stress. Fig. S3. Screening of DEGs in four lettuce varieties. Fig. S4. qRT-PCR validation of partial differential lettuce gene. Fig. S5. GO enrichment analysis of different expression genes from different cold tolerant lettuce material response to cold stress. Fig. S6. Partitioning Differential Genes into 27 Gene Modules Using WGCNA. Fig. S7. The temperature change during lettuce growth [file 12870_2024_5099_MOESM1_ESM.pdf]

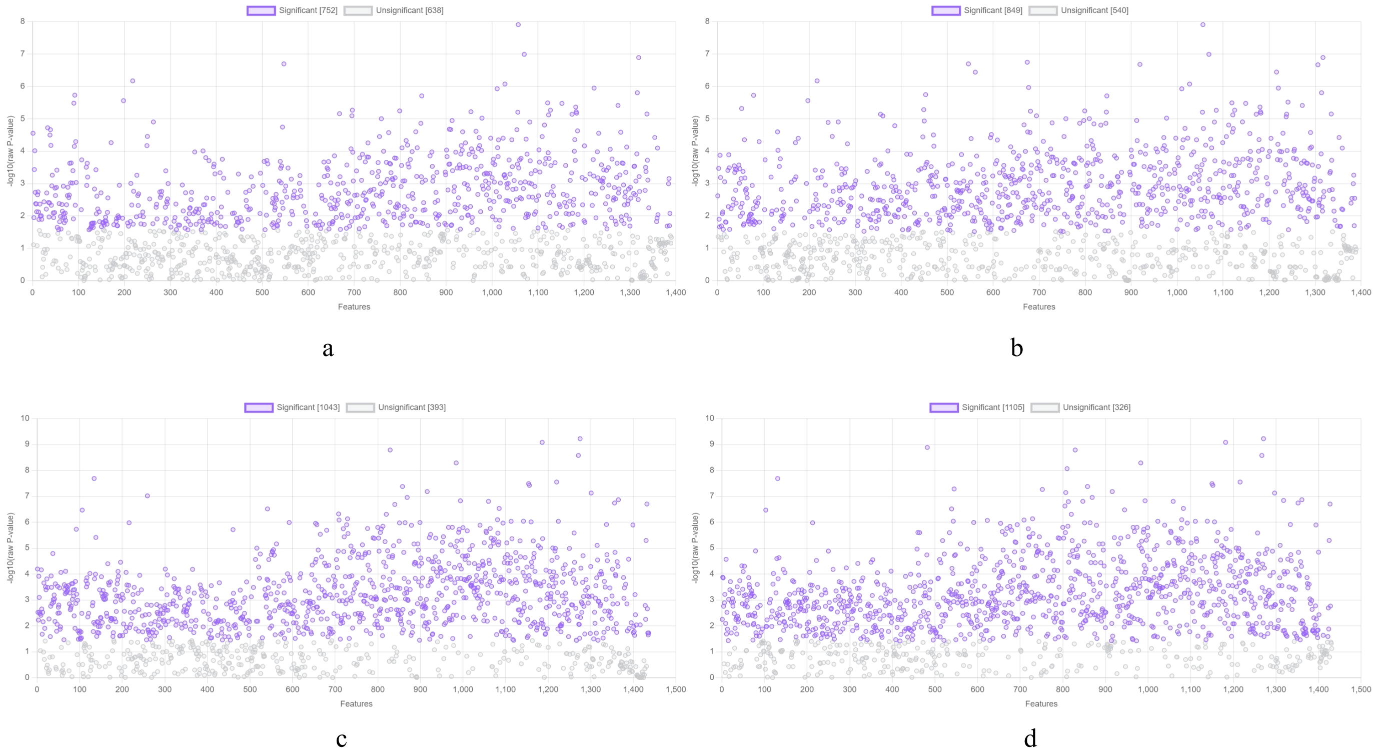

Fig. S1: Screening of metabolites of different cold tolerant lettuce material response to cold stress

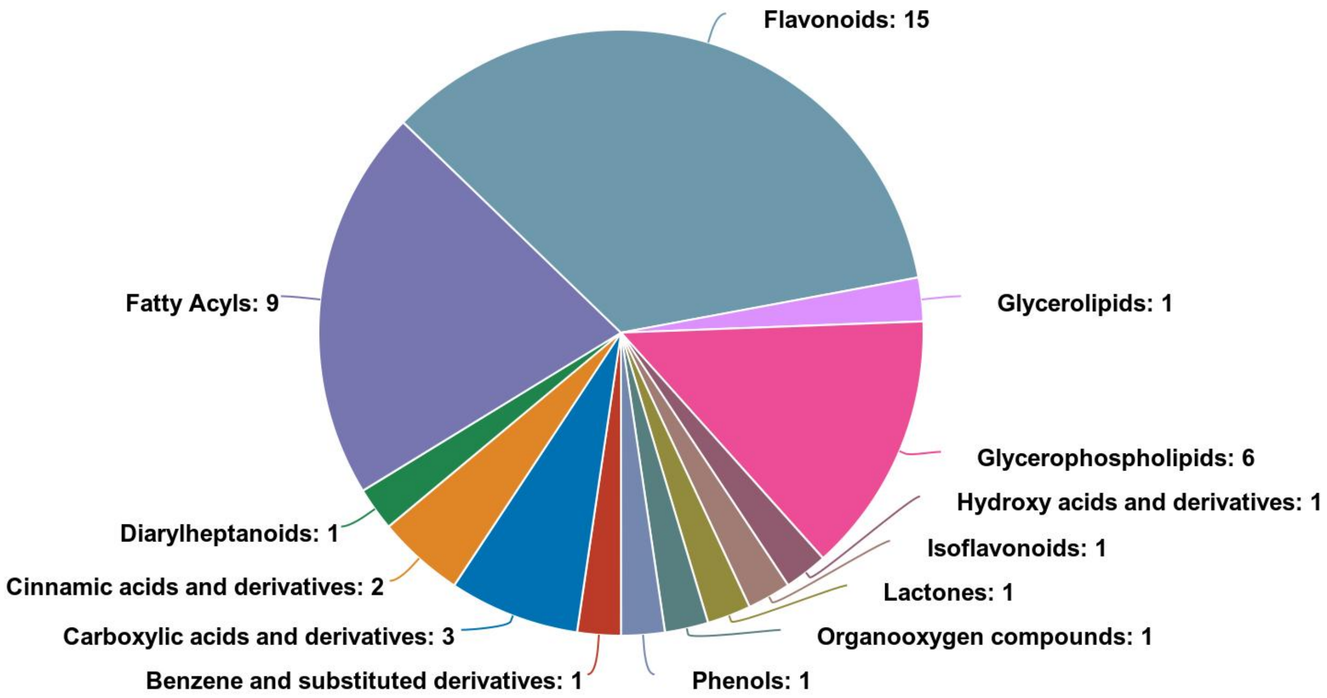

Fig. S2: Classification of 43 metabolites from different cold tolerant lettuce material response to cold stress

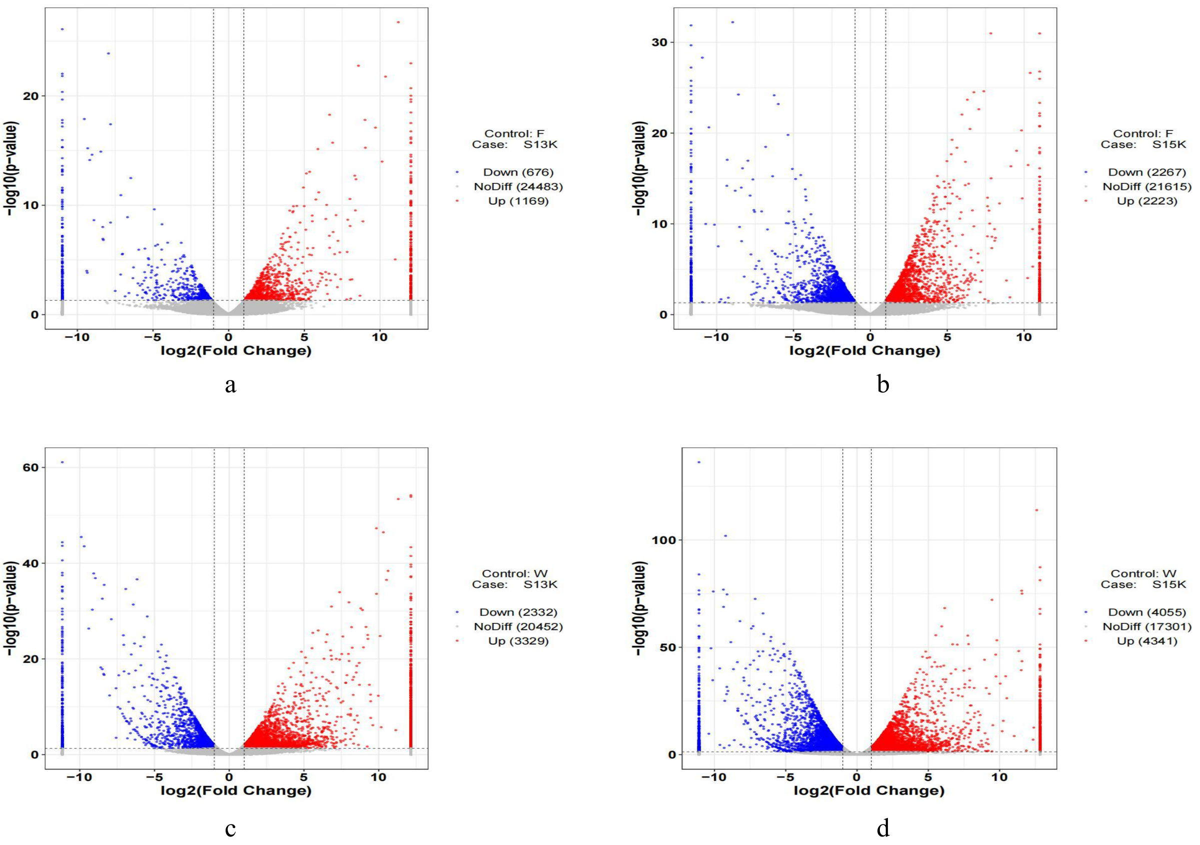

Fig. S3： Screening of DEGs in four lettuce varieties

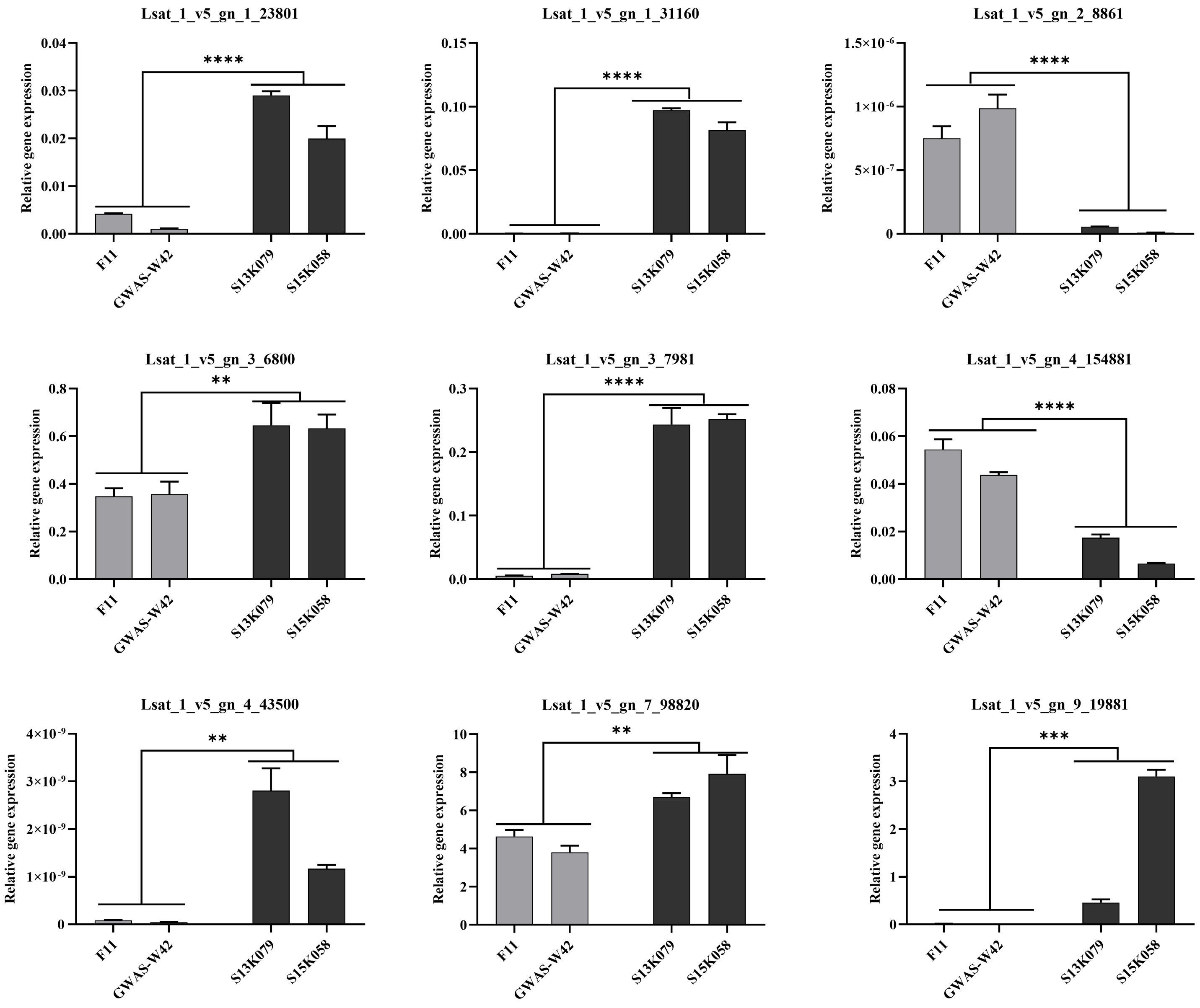

Fig. S4: qRT-PCR validation of partial differential lettuce gene

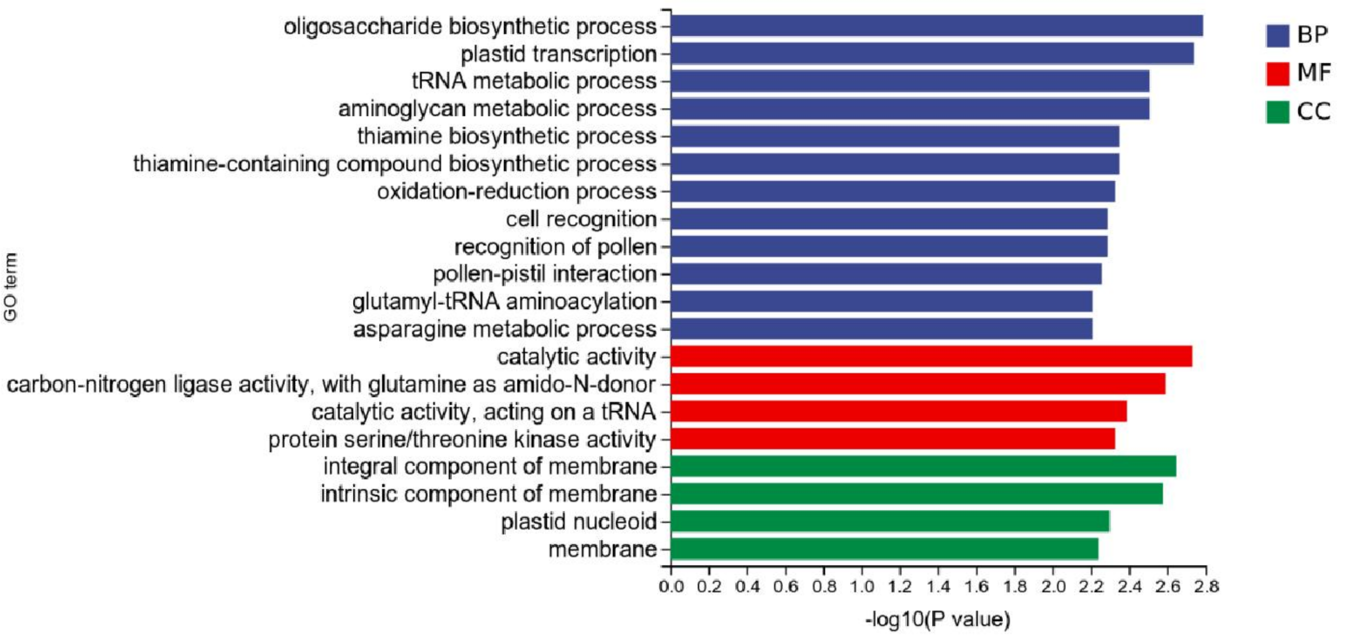

Fig. S5: GO enrichment analysis of different expression genes from different cold tolerant lettuce material response to cold stress

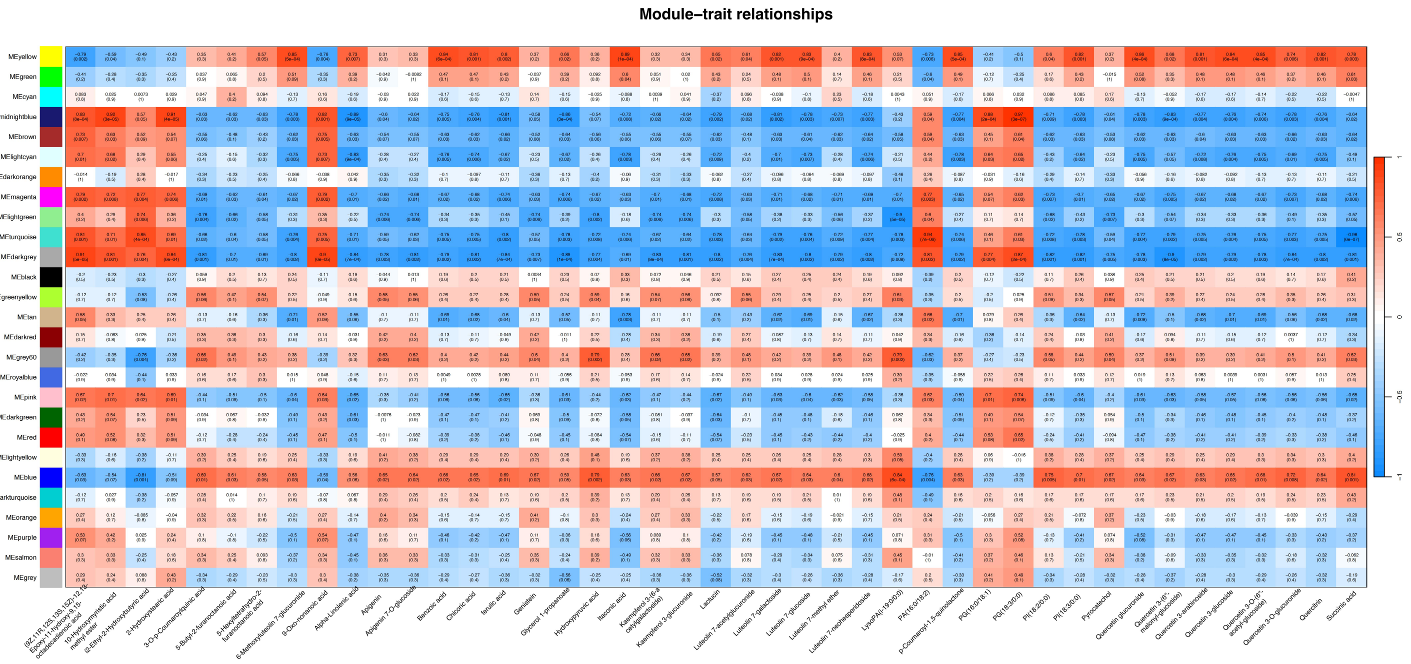

Fig. S6: Partitioning Differential Genes into 27 Gene Modules Using WGCNA

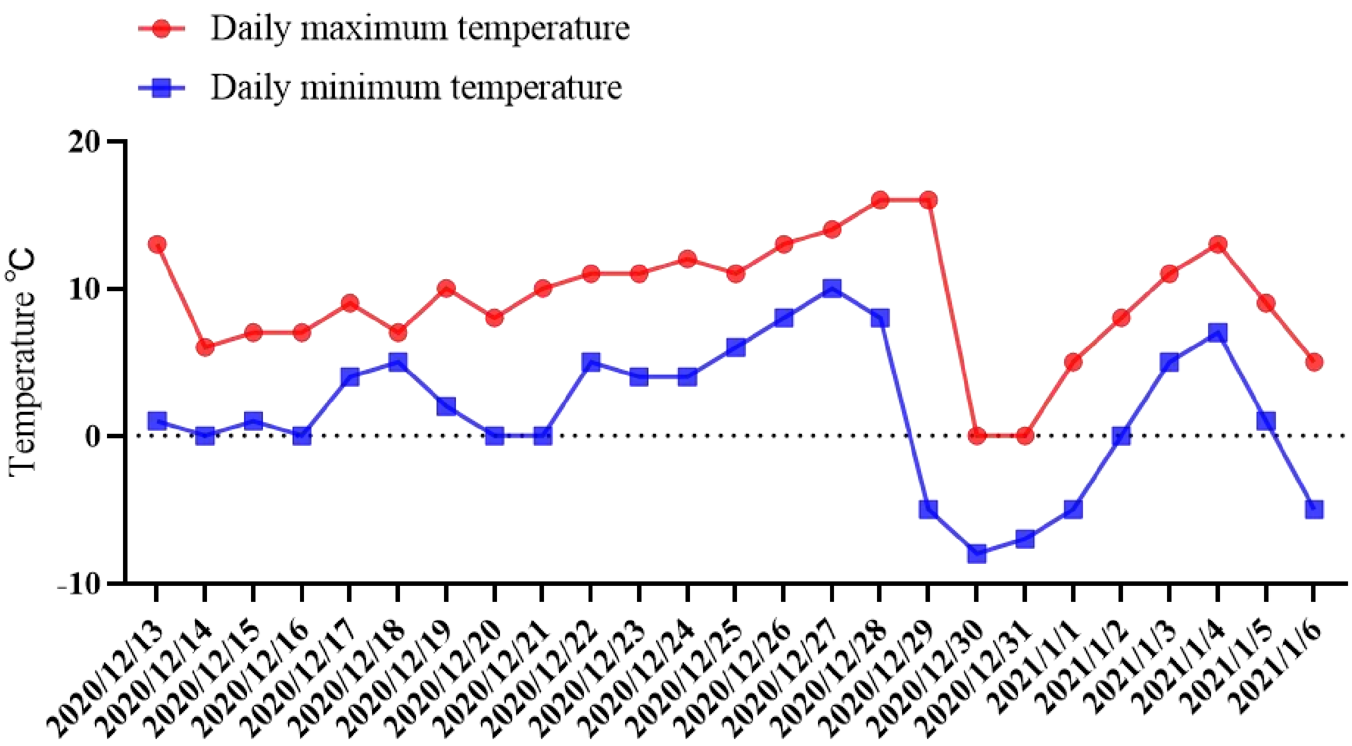

Fig. S7: The temperature change during lettuce growth
